# Dupilumab treatment reduces caregiver-reported skin pain in patients with moderate-to-severe atopic dermatitis aged 6 months to 5 years

**DOI:** 10.3389/fped.2024.1446779

**Published:** 2024-09-04

**Authors:** Amy S. Paller, Jonathan I. Silverberg, Mercedes E. Gonzalez, Lynda C. Schneider, Robert Sidbury, Zhen Chen, Ashish Bansal, Zhixiao Wang, Randy Prescilla

**Affiliations:** ^1^ Departments of Dermatology and Pediatrics, Northwestern University Feinberg School of Medicine, Chicago, IL, United States; ^2^ Division of Dermatology, Ann and Robert H. Lurie Children’s Hospital, Chicago, IL, United States; ^3^ Department of Dermatology, The George Washington University School of Medicine and Health Sciences, Washington, DC, United States; ^4^ Pediatric Skin Research, Coral Gables, FL, United States; ^5^ The Phillip Frost Department of Dermatology, University of Miami Miller School of Medicine, Miami, FL, United States; ^6^ Division of Immunology, Boston Children’s Hospital and Harvard Medical School, Boston, MA, United States; ^7^ Division of Dermatology, Department of Pediatrics, University of Washington School of Medicine and Seattle Children’s Hospital, Seattle, WA, United States; ^8^ Regeneron Pharmaceuticals Inc., Tarrytown, NY, United States; ^9^ Sanofi, Cambridge, MA, United States

**Keywords:** children, atopic dermatitis, skin pain, efficacy, dupilumab

## Abstract

**Background:**

Moderate-to-severe atopic dermatitis (AD) often has a profound impact on the quality of life of young children and their caregivers. One of the most burdensome symptoms reported by patients is skin pain.

**Methods:**

This *post hoc* analysis focuses on the impact of dupilumab treatment on skin pain in young children using data from the LIBERTY AD PRESCHOOL part B (NCT03346434), a 16-week randomized, double-blind, placebo-controlled, phase 3 study in 162 children aged 6 months to 5 years with moderate-to-severe AD receiving dupilumab or placebo, plus topical corticosteroids (TCS). Analyses were performed on the full analysis set and subgroups of patients who did not achieve an Investigator's Global Assessment score of 0 or 1 (IGA >1 subgroup), or who did not achieve a 75% improvement from baseline in the Eczema Area and Severity Index (<EASI-75 subgroup), at week 16 (patients who did not achieve the primary or key secondary endpoints in LIBERTY AD PRESCHOOL part B).

**Results:**

At week 16, change from baseline in the skin pain NRS was significantly greater in the dupilumab group vs. the placebo group (−3.93 vs. −0.62, *p* < 0.0001) and significantly more patients receiving dupilumab vs. placebo achieved a clinically meaningful improvement at week 16 (47.2% vs. 10.8%, *p* < .0001). Similar results between dupilumab vs. placebo were seen in the two subgroups IGA >1 and <EASI-75.

**Conclusions:**

This analysis showed rapid, clinically meaningful, and statistically significant improvements in skin pain in patients treated with dupilumab plus TCS vs. placebo plus TCS.

## Introduction

Atopic dermatitis (AD) is a chronic inflammatory systemic disease with an estimated prevalence of 12% in children younger than age 6 years (1). The most burdensome symptoms reported by patients are itch (pruritus) and skin pain, which can negatively impact the quality of life of both affected children and their caregivers and families (2, 3).

In contrast to itch, skin pain is less well-characterized in children with AD. However, its importance is increasingly being recognized when evaluating the burden of AD (4–7). A recent study investigating the burden and characteristics of skin pain among children with AD reported that nearly half the infants and children with AD had caregiver-reported skin pain, which was reported to be more intense with more severe disease (8). Furthermore, greater intensity of skin pain was associated with an overall worse quality of life (8).

Patient-reported outcomes can provide an important complement to clinician-reported outcomes in both clinical trials and daily clinical practice. The use of observer-reported outcomes (ObsROs) is supported when a patient (for example, a young child) is unable to provide a reliable and valid self-reported response about their own health experiences (9, 10). The caregiver-reported skin pain Numeric Rating Scale (NRS) is a validated ObsRO tool for the assessment of skin pain severity in children aged 6 months to 5 years with moderate-to-severe AD (11).

Phase 3 trials in children, adolescents, and adults with AD demonstrated that treatment with dupilumab, compared with placebo, leads to substantial improvements in AD signs, symptoms, and quality of life, with an acceptable safety profile (12–16).

In the LIBERTY AD PRE-SCHOOL part B study (NCT03346434), a phase 3 placebo-controlled trial, dupilumab administered with concomitant low-potency topical corticosteroids (TCS) significantly improved AD signs, symptoms, and quality of life in children aged 6 months to 5 years with moderate-to-severe AD (16).

This study evaluated the impact of treatment with dupilumab plus low-potency TCS on skin pain in children aged 6 months to 5 years with moderate-to-severe AD who had been included in the LIBERTY AD PRE-SCHOOL part B study.

## Methods

This was a *post hoc* analysis of data from LIBERTY AD PRESCHOOL part B (NCT03346434), a 16-week randomized, double-blind, parallel-group, placebo-controlled phase 3 study of dupilumab plus TCS in patients aged 6 months to 5 years with moderate-to-severe AD that was inadequately controlled with topical therapies (16).

The full study design and primary analysis of LIBERTY AD PRESCHOOL part B has been reported previously (16). Briefly, patients included were aged 6 months to 5 years at screening, with moderate-to-severe AD according to the consensus criteria of the American Academy of Dermatology (17) and a documented recent history (within 6 months before the screening visit) of inadequate response to topical AD medication. At screening, patients had an Investigators’ Global Assessment (IGA) score ≥3, Eczema Area and Severity Index (EASI) ≥16, body surface area (BSA) affected by AD ≥10%, and worst scratch/itch NRS score ≥4.

The primary and key secondary endpoints of LIBERTY AD PRESCHOOL part B were the proportion of patients achieving an IGA score of 0/1 (clear/almost clear skin) at week 16 and the proportion of patients achieving at least a 75% improvement from baseline in EASI (EASI-75) at week 16, respectively.

LIBERTY AD PRESCHOOL part B was conducted in accordance with the provisions of the Declaration of Helsinki, the International Conference on Harmonisation Good Clinical Practice guidelines, and applicable regulatory requirements. Local institutional review boards or ethics committees at each trial center oversaw trial conduct and documentation, and reviewed and approved the study protocol. Written informed consent was obtained from a parent or legal guardian for each study participant.

Patients received subcutaneous dupilumab every 4 weeks (200 mg for baseline bodyweight ≥5 to <15 kg; 300 mg for baseline bodyweight ≥15 to <30 kg) or matched placebo, with a standardized daily regimen of low-potency TCS (hydrocortisone acetate 1% cream). In-clinic visits were planned at baseline, week 1, week 2, and week 4, then monthly through week 16, with weekly telephone visits between clinic visits. The validated caregiver-reported skin pain NRS was used to evaluate patients’ AD-related skin pain during the previous 24 h on an 11-point scale ranging from 0 (no pain) to 10 (worst pain possible). A 2-to-4-point improvement in skin pain NRS is considered to be clinically meaningful in children aged 6 months to 5 years (11).

Analyses were performed in the full analysis set (FAS), which included all randomized patients, and in the subgroups of patients who did not achieve an IGA score of 0 or 1 (IGA >1) at week 16, or who did not achieve EASI-75 at week 16 (<EASI-75), (i.e., patients who did not achieve the primary or key secondary endpoints in LIBERTY AD PRESCHOOL part B).

Least squares (LS) mean change from baseline in skin pain NRS was analyzed using analysis of covariance, with treatment group, stratification factors, and relevant baseline measurements included in the model. Patients with missing values at week 16 due to rescue treatment, withdrawn consent, adverse events (AEs), or lack of efficacy (as deemed by the investigator) were imputed by worst observation carried forward. Missing values due to other reasons were imputed using multiple imputation.

The proportion of patients with ≥4-point improvement from baseline in skin pain NRS was analyzed using a Cochran-Mantel-Haenszel test after adjustment for randomization strata. Patients with missing values at week 16 due to use of rescue treatment, withdrawn consent, AEs, or lack of efficacy (as deemed by the investigator) were considered to be non-responders. Missing data due to any other reasons, including COVID-19, were imputed using multiple imputation.

Safety outcomes were assessed in the safety analysis set (SAS), which included all patients who received any study drug, and in the IGA >1 and <EASI-75 subgroups. Safety outcomes included proportions of patients with ≥1 treatment-emergent adverse event (TEAE), ≥1 serious TEAE, TEAEs leading to permanent study withdrawal, and patients with use of ≥1 rescue medication.

Statistical significance (*p* < 0.05) was calculated for dupilumab vs. placebo; all *p-*values were regarded as nominal, with the exception of change from baseline in skin pain NRS at week 16, which was a pre-specified secondary endpoint in LIBERTY AD PRESCHOOL part B.

All analyses were performed using SAS version 9.4 (Cary, NC, USA) or higher.

## Results

162 patients were included in the FAS (83 dupilumab plus TCS; 79 placebo plus TCS) (Table 1). In the dupilumab and placebo groups, 60 and 76 patients, respectively, did not achieve an IGA score <1 at week 16 and were included in the IGA >1 subgroup. 39 and 71 patients, for dupilumab and placebo, respectively, did not achieve EASI-75 and were included in the <EASI-75 subgroup. Baseline demographics and disease characteristics were balanced between the treatment groups and the IGA >1 and <EASI-75 subgroups.

**Table 1:** Baseline demographics and disease characteristics.

	FAS		IGA >1 subgroup		<EASI-75 subgroup	
	Dupilumab + TCS (*n* = 83)	Placebo + TCS (*n* = 79)	Dupilumab + TCS (*n* = 60)	Placebo + TCS (*n* = 76)	Dupilumab + TCS (*n* = 39)	Placebo + TCS (*n* = 71)
Age, mean (SD), years	3.9 (1.20)	3.8 (1.30)	4.00 (1.19)	3.80 (1.22)	4.01 (1.16)	3.77 (1.22)
≥6 months to <2 years, *n* (%)	6 (7.2)	5 (6.3)	4 (6.7)	4 (5.3)	2 (5.1)	4 (5.6)
≥2 years to <6 years, *n* (%)	77 (92.8)	74 (93.7)	56 (93.3)	72 (94.7)	37 (94.9)	67 (94.4)
Sex (male), *n* (%)	44 (53.0)	55 (69.6)	35 (58.3)	53 (69.7)	20 (51.3)	49 (69.0)
Age at AD disease onset, *n* (%)						
<6 months	50 (60.2)	57 (72.2)	33 (55.0)	55 (72.4)	19 (48.7)	52 (73.2)
≥6 months	33 (39.8)	22 (27.8)	27 (45.0)	21 (27.6)	20 (51.3)	19 (26.8)
Duration of AD, mean (SD), years	3.4 (1.3)	3.4 (1.3)	3.4 (1.32)	3.5 (1.26)	3.3 (1.31)	3.4 (1.27)
Disease characteristics						
Patients with IGA score 3, *n* (%)	20 (24.1)	17 (21.5)	6 (10.0)	15 (19.7)	5 (12.8)	13 (18.3)
Patients with IGA score 4, *n* (%)	63 (75.9)	62 (78.5)	54 (90.0)	61 (80.3)	34 (87.2)	58 (81.7)
EASI, mean (SD)	35.1 (13.88)	33.1 (12.18)	37.6 (14.20)	33.2 (12.32)	37.2 (14.95)	33.3 (12.45)
Skin pain, mean (SD)	6.8 (1.76)	7.2 (1.84)	6.9 (1.86)	7.1 (1.85)	7.1 (1.90)	7.1 (1.89)
Worst scratch/itch NRS, mean (SD)	7.5 (1.32)	7.6 (1.49)	7.6 (1.34)	7.6 (1.49)	7.8 (1.37)	7.6 (1.52)
SCORAD, mean (SD)	72.7 (12.95)	72.2 (11.44)	75.3 (11.77)	72.4 (11.62)	75.0 (12.92)	72.7 (11.71)
BSA involvement	59.3 (22.5)	57.4 (20.9)	62.3 (21.91)	57.3 (21.01)	59.5 (22.56)	56.5 (21.27)

AD, atopic dermatitis; BSA, body surface area; EASI, Eczema Area and Severity Index; EASI-75, 75% improvement from baseline in EASI; FAS, full analysis set; IGA, Investigator's Global Assessment; NRS, Numerical Rating Scale; SCORAD, SCORing Atopic Dermatitis; SD, standard deviation; TCS, topical corticosteroids.

**Figure 1:**
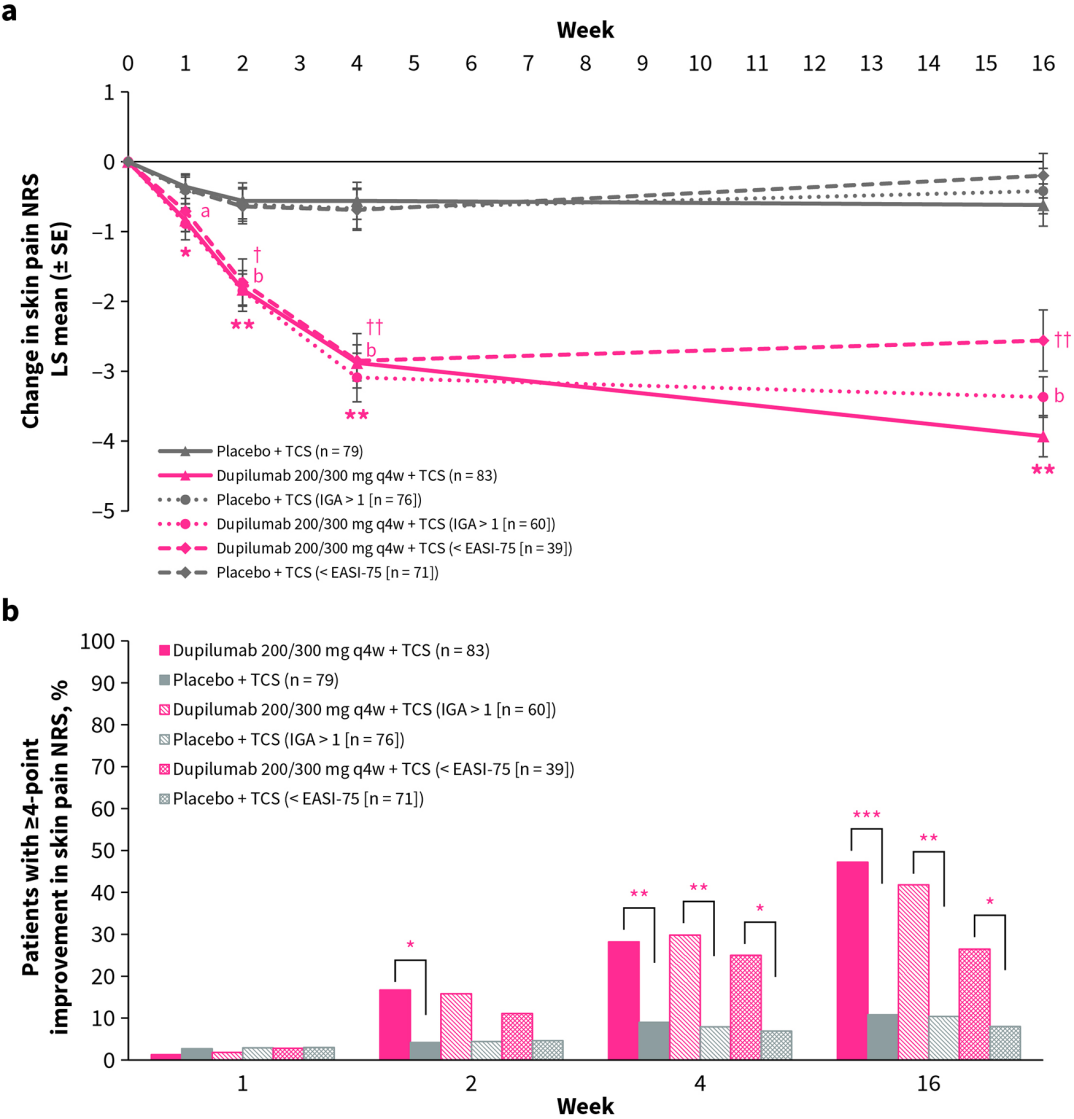
**(a)** LS mean change from baseline in skin pain NRS over time for dupilumab vs. placebo. *p < 0.05, **p < 0.0001 (FAS); ^a^*p* < 0.05, ^b^*p* < 0.0001 (IGA >1); ^†^*p* < 0.01, ^††^*p* < 0.0001 (<EASI-75). **(b)** Proportion of patients with ≥4-point improvement in skin pain NRS over time for dupilumab vs. placebo. *p < 0.05, **p < 0.01, ***p < 0.0001. EASI-75, 75% improvement from baseline in Eczema Area and Severity Index; IGA, Investigator's Global Assessment; NRS, Numerical Rating Scale; q4w, every 4 weeks; SE, standard error; TCS, topical corticosteroids.

At week 16, LS mean change from baseline in skin pain NRS was significantly greater in the dupilumab group compared with the placebo group (−3.93 vs. −0.62, *p* < 0.0001, respectively), with a significant benefit for dupilumab compared with placebo evident from week 1 onward (Figure 1a). In the subgroup of patients with IGA >1 (60 in the dupilumab group; 76 in the placebo group) and in the subgroup of patients with <EASI-75 (39 in the dupilumab group; 71 in the placebo group) at week 16, a significant benefit for dupilumab compared with placebo was seen from week 2 onward, maintained through week 16 (−3.37 vs. −0.42 and −2.56 vs. −0.20, respectively, both *p* < 0.0001; Figure 1a).

At week 16, the proportion of patients with ≥4-point improvement from baseline in skin pain NRS was significantly greater in the dupilumab group compared with the placebo group (47.2% vs. 10.8%, *p* < 0.0001, respectively), with a significant benefit for dupilumab compared with placebo being evident from week 1 onward (Figure 1b). In the subgroup of patients with IGA >1 and the subgroup of patients with <EASI-75 at week 16, a significant benefit for dupilumab compared with placebo was seen from week 4 until week 16 (41.8% vs. 10.4% and 26.5% vs. 8.0% respectively, both *p* < 0.05; Figure 1b).

The primary analysis of the LIBERTY AD PRE-SCHOOL part B study reported that dupilumab was generally well tolerated, with an acceptable safety profile (16). Safety outcomes for patients in the SAS and the IGA >1 and <EASI-75 subgroups were comparable (Table 2) (16, 18). In the SAS and in both subgroups, the proportion of patients with ≥1 TEAE and the use of ≥1 rescue medication was lower in the dupilumab groups compared with the placebo groups (Table 2) (16, 18). Serious TEAEs (exacerbation of AD) only occurred in the placebo groups, and there was only one occurrence of TEAEs leading to permanent withdrawal in both the dupilumab and placebo groups (Table 2) (16, 18).

**Table 2:** Number of patients with AEs reported in the safety analysis set^16^ and IGA >1^18^ and >EASI-75 subgroups, at week 16.

	SAS^a^		IGA >1 subgroup		<EASI-75 subgroup	
	Dupilumab + TCS (*n* = 83)	Placebo + TCS (*n* = 78)	Dupilumab + TCS (*n* = 60)	Placebo + TCS (*n* = 75)	Dupilumab + TCS (*n* = 39)	Placebo + TCS (*n* = 70)
Patients with ≥1 TEAE, *n* (%)	53 (63.9)	58 (74.4)	39 (65.0)	55 (73.3)	27 (69.2)	51 (72.9)
TEAEs reported in ≥3% of patients, *n* (%)^a^						
Infections and infestations	35 (42.2)	40 (51.3)	25 (41.7)	37 (49.3)	17 (43.6)	33 (47.1)
Nasopharyngitis	7 (8.4)	7 (9.0)	4 (6.7)	6 (8.0)	3 (7.7)	4 (5.7)
Upper respiratory tract infection	5 (6.0)	6 (7.7)	3 (5.0)	5 (6.7)	2 (5.1)	5 (7.1)
Impetigo	3 (3.6)	6 (7.7)	2 (3.3)	6 (8.0)	2 (5.1)	5 (7.1)
Skin and subcutaneous tissue disorders	17 (20.5)	28 (35.9)	14 (23.3)	28 (37.3)	11 (28.2)	25 (35.7)
Exacerbation of AD	11 (13.3)	25 (32.1)	9 (15.0)	25 (33.3)	7 (17.9)	23 (32.9)
Urticaria	1 (1.2)	4 (5.1)	1 (1.7)	4 (5.3)	0	3 (4.3)
Respiratory, thoracic, and mediastinal disorders	9 (10.8)	15 (19.2)	6 (10.0)	15 (20.0)	5 (12.8)	13 (18.6)
Asthma	3 (3.6)	5 (6.4)	2 (3.3)	5 (6.7)	1 (2.6)	4 (5.7)
Cough	0	5 (6.4)	0	5 (6.7)	0	5 (7.1)
Blood and lymphatic system disorders	6 (7.2)	7 (9.0)	5 (8.3)	7 (9.3)	3 (7.7)	7 (10.0)
Lymphadenopathy	3 (3.6)	6 (7.7)	2 (3.3)	6 (8.0)	1 (2.6)	6 (8.6)
General disorders and administration site conditions	5 (6.0)	9 (11.5)	3 (5.0)	9 (12.0)	0	9 (12.9)
Pyrexia	1 (1.2)	7 (9.0)	1 (1.7)	7 (9.3)	0	7 (10.0)
Patients with ≥1 serious TEAE, *n* (%)^b^	0	4 (5.1)	0	3 (4.0)	0	3 (4.3)
Patients with TEAE leading to dose withdrawal permanently, *n* (%)	1 (1.2)^c^	1 (1.3)^d^	1 (1.7)^c^	1 (1.3)^d^	1 (2.6)^c^	1 (1.4)^d^
Patients with ≥1 rescue medication, *n* (%)^e^	16 (19.3)	49 (62.8)	16 (26.7)	49 (65.3)	16 (41.0)	49 (70.0)

AD, atopic dermatitis; AE, adverse event; EASI-75, 75% improvement from baseline in Eczema Area and Severity Index; IGA, Investigator's Global Assessment; MedDRA, Medical Dictionary for Regulatory Activities; SAS, safety analysis set; TCS, topical corticosteroids; TEAE, treatment-emergent adverse effect.

^a^ According to primary MedDRA System Organ Class, and Preferred Term.

^b^ The only serious TEAEs, which occurred exclusively in the placebo groups, were exacerbation of AD.

^c^ Exacerbation of AD.

^d^ Nightmares due to blood withdrawal.

^e^ Medium- or high-potency TCS, systemic corticosteroids, non-steroidal immunosuppressants (e.g., cyclosporine, methotrexate, mycophenolate mofetil, azathioprine), crisaborole, or topical calcineurin inhibitors could be provided to study patients as rescue medication after day 14.

## Discussion

Skin pain is a symptom frequently reported by patients and is increasingly being recognized when evaluating the burden of AD (4–8). While associated with reduced quality of life, skin pain is not well characterized in children (8). Recently, the importance of exploring pain in AD as an independent symptom has been underscored by the fourth international consensus meeting to harmonize core outcome measures for atopic eczema/dermatitis clinical trials (the HOME initiative) (19).

In children aged 6 months to 5 years with moderate-to-severe AD, dupilumab treatment with concomitant low-potency TCS compared with placebo plus TCS provided rapid and significant improvements in caregiver-reported skin pain, with improvements sustained over 16 weeks. Dupilumab plus TCS also provided a rapid and clinically meaningful response of at least a 4-point improvement in skin pain NRS scores in significantly more patients treated with dupilumab compared with placebo, sustained over 16 weeks. Significant benefits for dupilumab compared with placebo were seen in the FAS, and in the subgroup of patients with IGA >1 and the subgroup of patients with <EASI-75 at week 16. These results confirm and extend earlier findings demonstrating the efficacy of dupilumab treatment in children aged 6 months to 5 years with moderate-to-severe AD (16) and are in line with previously reported results for adults with moderate-to-severe AD (20).

The clinician-reported endpoints, achievement of IGA 0/1 or EASI-75, are valuable measures that are widely used in clinical trials to define an optimal response to therapy. However, these endpoints do not capture the impact from the patients’ perspective, including of treatment for skin pain (19). This analysis shows significant and potentially clinically meaningful improvements in caregiver-reported skin pain in the dupilumab-treated subgroups, even in patients who did not achieve IGA 0/1 or EASI-75 at week 16.

This analysis provides support that includes the use of tools, such as the skin pain NRS, to capture the caregiver and/or patient perspective of the benefits of therapy may provide a more holistic view of treatment response. Considering the multidimensional nature of AD, assessing skin pain could be of value to prescribing physicians in their routine clinical practice. However, it should be noted that it may be challenging for caregivers to assess skin pain and distinguish it from itch, especially in young, non-verbal children (21).

Strengths of this study include the randomized, placebo-controlled study design. Limitations of the study include the relatively small number of children in the youngest age range (6 months to <2 years), the short (16-week) study duration, and the *post hoc* nature of this analysis including nominal *p-*values.

In conclusion, dupilumab treatment with concomitant low-potency TCS provided clinically meaningful and rapid, statistically significant improvements vs. placebo in skin pain in children aged 6 months to 5 years with moderate-to-severe AD.

## Data Availability

The data analyzed in this study is subject to the following licenses/restrictions: Qualified researchers may request access to study documents (including the clinical study report, study protocol with any amendments, blank case report form, statistical analysis plan) that support the methods and findings reported in this manuscript. Individual anonymized participant data will be considered for sharing once the indication has been approved by a regulatory body, if there is legal authority to share the data and there is not a reasonable likelihood of participant re-identification. Requests to access these datasets should be directed to https://vivli.org/.
